# Yellow Emissive Tris(8-hydroxyquinoline) Aluminum by the Incorporation of ZnO Quantum Dots for OLED Applications

**DOI:** 10.3390/mi12101173

**Published:** 2021-09-29

**Authors:** Aya Hekmet Makki, Si-Hyun Park

**Affiliations:** 1 Department of Electronics Engineering, Yeungnam University, Gyeongsan 38541, Korea; ayah@ynu.ac.kr; 2 Laser and Optoelectronics Engineering Department, University of Technology-Iraq, Baghdad 10066, Iraq

**Keywords:** OLED, Alq3, Alq3-ZnO nanohybrids

## Abstract

Tris(8-hydroxyquinoline) aluminum complexes are of significant interest because of their remarkable optical and electrical properties, both as an emissive layer and electron injection layer. They emit light in the blue and green ranges of the visible spectrum, so for white organic light emitting diodes (OLEDs), yellow emission is required as well. In this study, we propose the use of zinc oxide quantum dots to tune the emission color of the complex while maintaining its luminous efficiency. Hence, tris(8-hydroxyquinoline) aluminum-zinc oxide nanohybrids with different zinc oxide quantum dots concentrations (10, 20, or 30 wt.%) were synthesized. The structural properties were characterized using powder X-ray diffraction analysis, while the composition and optical characteristics were characterized by Fourier transform infrared spectroscopy, UV-visible absorption spectroscopy, and photoluminescence emission spectroscopy. The results show that increased levels of zinc oxide quantum dots lead to a decrease in crystallinity, double hump emission and a slight red shift in emission peaks. Also, at 20 and 30 wt.% of zinc oxide quantum dots concentrations, yellow emission was observed.

## 1. Introduction

Organic light-emitting diodes (OLEDs) have attracted significant research attention, especially for next-generation flexible and foldable devices compared with their inorganic counterparts, owing to their outstanding material properties, such as high flexibility, uniform emission over a large area, tunable wavelength, ease of fabrication, low cost and power consumption, and environmental friendliness [1,2,3,4,5]. Throughout the last couple of years, the study has therefore focused on organic-electroluminescent materials. Among those materials, Tris (8-hydroxyquinoline) aluminum (III) (Alq3) has been attracted and seen as a promising candidate for its excellent electrical transport and emission properties, as well as its high thermal stability. As a result, it is a better choice for emissive and electron-transparent layers in OLED and organic light-emitting transistor (OLET) devices [6,7,8].

Alq3 is an organometallic semiconductor material which exists in several crystalline phases (α, β, γ, δ, and ε) depending on the synthesis method. Previous studies have shown blue fluorescence in δ- and γ phases while green fluorescence was found in the other phases under UV excitation [9,10,11].

Color tuning in Alq3 was achieved previously by chemical means in which the band gap, and therefore complex emission wavelength, was tailored by the attachment of electron-donating or electron withdrawing substituents to the quinolinolate ligand [12,13].

The only drawback to Alq3 complexes is their high environmental sensitivity and ease of photooxidation, which results in low stability and hence luminescence efficiency degradation, subsequently limiting their use in OLED devices [14]. Many researchers have proposed the use of hybrid (organometallic/inorganic) composites to overcome these issues. Besides, those hybrids showed significant improvements in mechanical strength, thermal stability, luminescent efficiency, and charge mobility when compared to pure Alq3 [15,16]. Cuba et al. [17] showed that Alq3-ZnO composites exhibited enhanced luminescence properties and a slight shift from green emission to greenish blue. Li et al. [18]. reported that Alq3-nano-TiO2 using 8-vinyl POSS as a modifier resulted in an enhancement in the photoluminescence (PL) and electroluminescence (EL) properties of the composites. In this study, we report the yellow emission of Alq3 upon the introduction of ZnO quantum dots (QDs) as a dopant and studied the effect of ZnO QDs concentration on the structural and optical properties of the samples. Alq3-ZnO nanohybrids were synthesized using varying concentrations of ZnO QDs (10, 20, and 30 wt.%).

## 2. Materials and Methods

### 2.1. Materials

Multi-wall carbon nanotubes (MWCNTs, 98%, Sigma Aldrich, Seoul, Korea), zinc acetate dihydrate (Zn(OAc)_2_∙2H_2_O, 98%, Duksan, Seoul, Korea), hydrochloric acid (HCl, 35%, Duksan, Seoul, Korea), Sulfuric acid (H_2_SO_4_, 98%, Duksan, Seoul, Korea), ethanol (CH_3_CH_2_OH, 99.9%, Duksan, Seoul, Korea), 8-hydroxyquinoline (99%, Junsei Chemical, Tokyo, Japan), aluminum chloride (AlCl_3_, 99%, Fluka, Seoul, Korea), and potassium hydroxide (KOH, 85%, DAEJUNG, Seoul, Korea). All chemicals were used as received without any further purification.

### 2.2. Methods

#### 2.2.1. Synthesis of ZnO QDs

Yellow emissive ZnO QDs were synthesized according to the procedure described by Yang et al. [19]. Multi-walled carbon nanotubes (MWCNTs) were functionalized with acidic treatment to produce functional MWCNTs (FMWCNTs). A 20 mg sample of FMWCNTs were suspended in ethanol (20 mL) with sonication for 20 min. A 6 mL aliquot was taken from suspended FMWCNT solution and added to an ethanolic zinc acetate dihydrate solution (0.09 M) and stirred at room temp for 30 min, followed by refluxing at 70 °C for 4 h. The solution was subsequently cooled and sonicated for 30 min, and then the supernatant containing suspended ZnO QDs was collected. The QDs were collected by centrifugation, cleaned several times with ethanol and water, and dried in an oven at 60° at atmospheric pressure.

#### 2.2.2. Synthesize of Alq3

Alq3 was synthesized as follows: 0.3 M of ethanolic 8-hydroxyquinoline solution was mixed with a 0.22 M AlCl_3_ ethanolic solution and the pH was neutralized using a KOH solution. The resulting mixture was refluxed at 70 °C for 5 h with stirring. The reaction mixture was subsequently allowed to cool to room temperature, and the yellow Alq3 precipitate was collected by centrifugation, washed with ethanol, and deionized water, and dried under vacuum at 100 °C for 12 h.

#### 2.2.3. Synthesis of Alq3-ZnO Nanohybrids

To synthesize Alq3-10 wt.% ZnO QDs, Alq3 (180 mg) was suspended in ethanol (32.5 mL) with stirring. ZnO QDs (20 mg) were also suspended in ethanol (3.25 mL) and sonicated. After 15 min, both suspensions were added with stirring and heated to 60 °C for 4 hrs. A precipitate was formed upon cooling which was collected by centrifugation and decantation, washed with ethanol, deionized water and dried under vacuum at 100 °C for 12 h. Identical procedures were used for the synthesis of Alq3-20 wt.% ZnO QD and Alq3-30 wt.% ZnO QD. For characterization, the MPD (Panalytical, Malvern, United Kingdom) Xpert Multipurpose X-ray Diffraction System was used for powder X-ray diffraction (XRD) analysis. A Spectrum 100 spectrometer (Perkin-Elmer, Waltham, Massachusetts, United States) and a Cary 5000 UV-vis-NIR spectrophotometer (Agilent Technologies, Santa Clara, California, United States) were used to record the Fourier transform infrared (FTIR) and absorption spectra, respectively.

## 3. Results and Discussion

**Figure 1:**
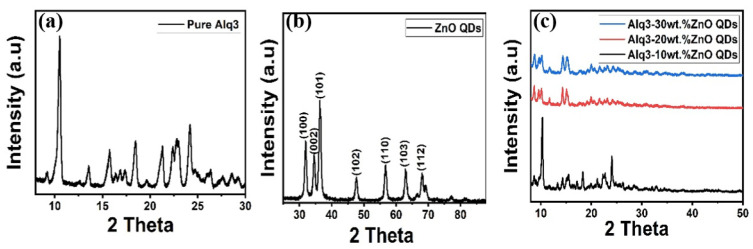
XRD pattern of (**a**) pure Alq3, (**b**) ZnO QDs and (**c**) Alq3-ZnO nanohybrids.

Figure 1 illustrates the structural properties of the synthesized Alq3, ZnO QDs, and Alq3-ZnO nanohybrids studied by XRD. The distinctive peaks of Alq3 and hexagonal ZnO observed in the XRD spectra are in agreement with the stated values standard (JCPDS 26-1550) and (JCPDS 75-0576), respectively. The crystallite sizes of the ZnO QDs were estimated using the Debye-Sherrer equation [20] and found to be 8.78, 8.85, 10.67, 11.08, 9.6, 10.41, and 10.04 nm for the (100), (002), (101), (102), (110), (103), and (112) crystalline phases, respectively. Figure 1c shows the diffraction pattern of Alq3-ZnO nanohybrids at different concentration levels of ZnO QDs. Increasing in ZnO QDs concentration results in a slight decrease in peaks intensity as well as a slight shifting of the diffraction peaks toward lower diffraction angles which confirms the incorporation of the ZnO QDs in the Alq3 matrix, resulting in positional rearrangement in the Alq3 lattice. In addition, no individual ZnO QDs peaks were observed for all the samples.

**Figure 2:**
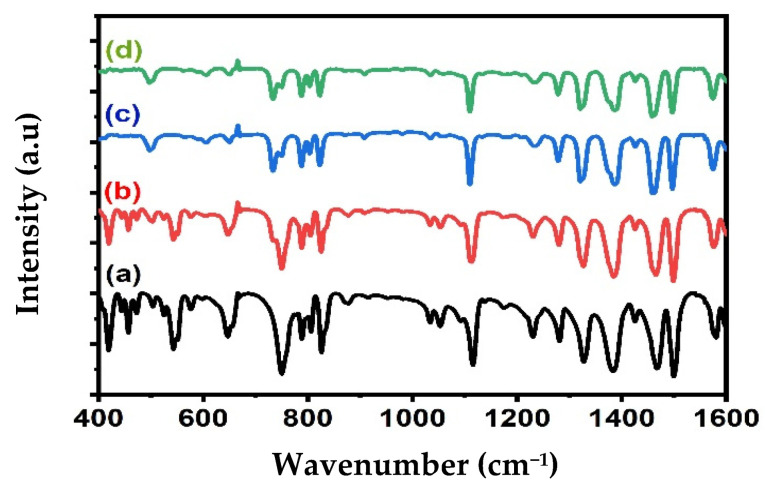
FTIR spectrum of (**a**) pure Alq3, (**b**) Alq3-10 wt.% ZnO QDs (**c**) Alq3-20 wt.% ZnO QDs (**d**) Alq3-30 wt.% ZnO QDs.

The composition of the Alq3 and Alq3-ZnO QD nanohybrids was studied by FTIR spectroscopy as shown in Figure 2. The FTIR spectrum of Alq3 shows all the expected characteristic peaks, as shown in Table 1. Doping with ZnO QDs resulted in weakening of the absorbance band in the 400–600 cm^−1^ region and shifting of several absorbance peaks. These observations are an indication of the interactions between the ZnO QDs and Alq3 and are consistent with the results of the XRD analysis. Notably, an absorption band around 500 cm^−1^ broadened with increasing ZnO QD concentration, which was attributed to Zn-O vibrations.

**Figure 3:**
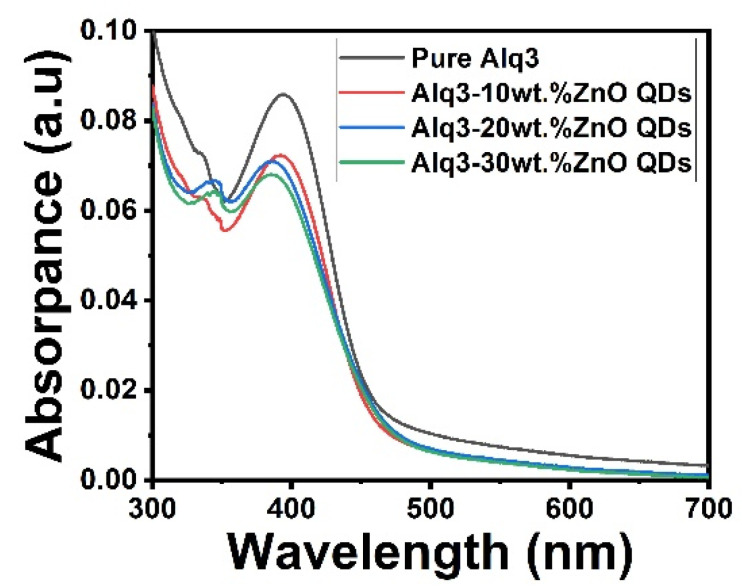
UV-Vis absorption spectrum of pure Alq3 and Alq3-ZnO nanohybrids.

**Figure 4:**
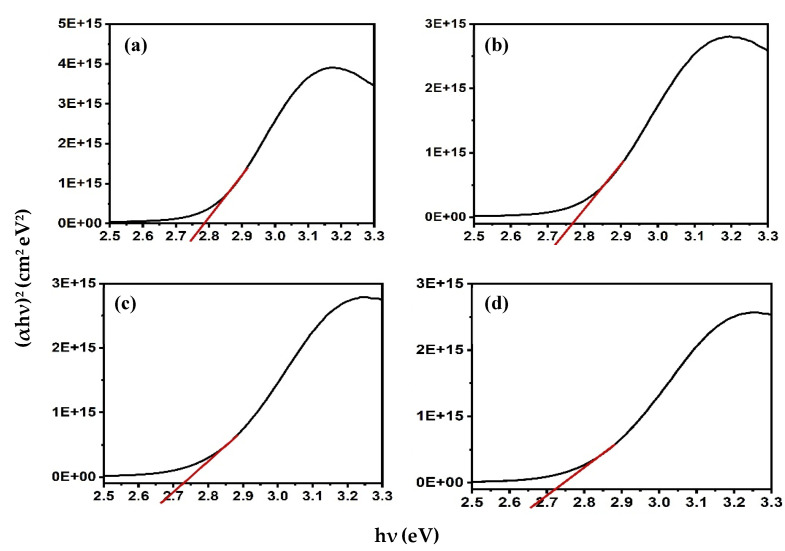
(αhν)^2^ versus hν curves for (**a**) pure Alq3, (**b**) Alq3-10 wt.% ZnO QDs (**c**) Alq3-20 wt.% ZnO QDs (**d**) Alq3-30 wt.% ZnO QDs.

The absorption spectra of Alq3 and Alq3-ZnO nanohybrids are shown in Figure 3. The spectrum of Alq3 showed an absorption peak at approximately 400 nm corresponding to the π–π* electronic transitions of the quinolinolate ligands [17]. Doping with ZnO QDs resulted in the emergence of an additional peak at approximately 350 nm which corresponded to the direct band gap transition of ZnO QDs [21]. The band gap energy of the samples was estimated using the Tauc method [22] with the following equation:

*αhv* = (*hv* − *Eg*)*^n^*, (1)

where *n* is 1/2 for allowed direct transitions, and 2 for allowed indirect transitions [22]. The direct band gap was estimated to be 2.79 eV for Alq3, which is consistent with the previously reported value [22]. The addition of ZnO QDs to Alq3 results in a slight decrease in the band gap with increasing concentration. The calculated bandgap energies of the Alq3-ZnO nanocomposites were calculated to be 2.76, 2.73, and 2.72 eV for Alq3-10 wt.% ZnO QDs, Alq3-20 wt.% ZnO QDs, and Alq3-10 wt.% ZnO QDs, respectively (Figure 4).

**Figure 5:**
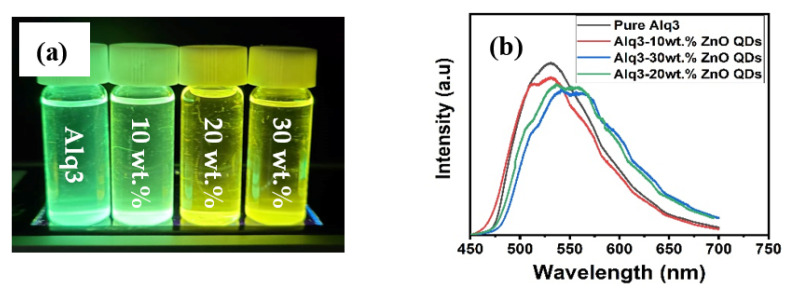
(**a**) Flurecence of pure Alq3 and Alq3-ZnO nanohybrids under UV excitation (**b**) PL emission spectrum.

**Figure 6:**
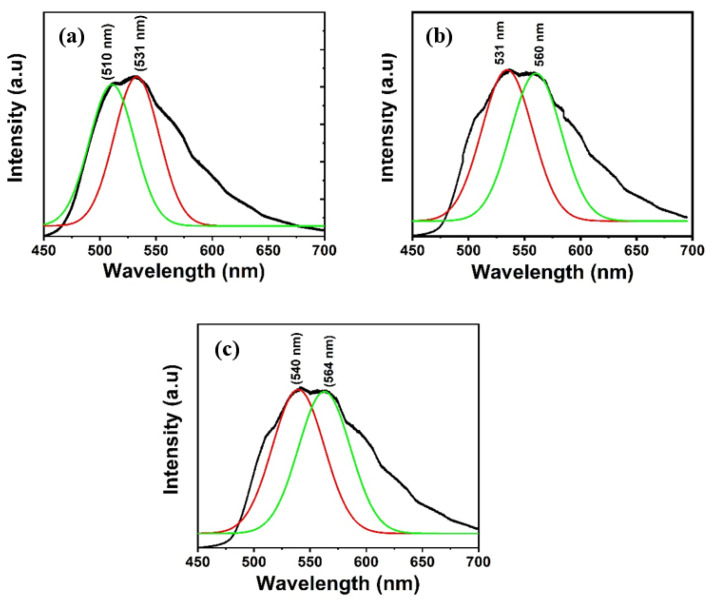
Deconvolution of the emission spectra of Alq3-ZnO nanohybrids. (**a**) Alq3-10 wt.% ZnO QDs (**b**) Alq3-20 wt.% ZnO QDs (**c**) Alq3-30 wt.% ZnO QDs.

The PL spectra of Alq3 and Alq3-ZnO nanohybrids after excitation with a He-Cd laser (325 nm) are shown in Figure 5. Pure Alq3 exhibits green emission centered around 525 nm. The presence of ZnO QDs results in introducing a new energy sate within the energy band gap of the Alq3 and hence results in a double-hump emission, the first due to the Alq3 molecules and the second due to the presence of ZnO QDs. Additionally, the emission peaks gradually red shifted with increasing ZnO QD concentration. The integrated PL intensity was found to be almost identical for all samples. The deconvoluted emission spectra are shown in Figure 6. The emission hump at approximately 520 nm correspond to Alq3, while the emission hump at approximately 560 nm corresponds to the ZnO QDs.

## 4. Conclusions

A yellow emissive Alq3 is obtained through physical means by doping with ZnO QDs. The Alq3-ZnO nanohybrids containing 10, 20, and 30 wt.% ZnO QDs were synthesized. Pure Alq3 exhibits green emission at 530 nm under excitation at 325 nm while doping with ZnO QDs results in a yellow emission peak centered at 560 nm for Alq3-20 wt.% ZnO QDs and Alq3-30 wt.% ZnO QDs. Therefore, based on our findings the color tuning in Alq3 can be achieved using ZnO QDs while maintaining the intrinsic PL intensity. This study demonstrates that these nanohybrids can be considered as promising candidates for use as emitting and transparent layers in yellow and white emissive OLEDs and OLETs.

## Figures and Tables

**Table 1:** IR Vibrational modes in FTIR spectra of pure Alq3 and Alq3-ZnO nanohybrids [3].

Wavenumber (cm^−1^)					Wavenumber (cm^−1^)				
Alq3	Alq3-10 wt.%	Alq3-20 wt.%	Alq3-30 wt.%	Assignment	Alq3	Alq3-10 wt.%	Alq3-20 wt.%	Alq3-30 wt.%	Assignment
419	419	420	422	Al-N vibration	1053	1053	1056	1056	C-O stretching
444	444	444	445	Al-O stretching	1115	1113	1110	1110	C-O stretching
457	457	459	459	Al-O stretching	1138	1137	1133	1133	C-O stretching
473	473	473	473	Al-O stretching	1175	1173	1173	1173	C-O stretching
504	503	497	497	Al-O stretching	1231	1231	1236	1232	C-O stretching
543	543	545	545	Al-O stretching	1281	1280	1278	1278	Aromatic amine
576	576	576	576	C-H bending	1327	1327	1321	1321	Aromatic amine
599	601	605	606	C-H bending	1383	1384	1386	1278	Aromatic amine
647	648	651	651	C-H bending	1425	1425	1426	1321	C=C stretching
750	750	733	733	C-H bending	1468	1466	1460	1385	C=C stretching
788	788	788	187	C-H bending	1499	1499	1497	1497	C=C bending
806	805	803	803	C-H bending	1580	1576	1575	1575	C=C bending
877	877	823	823	C-H bending	1605	1604	1603	1603	C=C bending
1034	1034	1034	1034	C-O stretching					

## Data Availability

The data used to support the findings of this study are included within the article.
